# *Bacillus cereus* Improves Performance of Brazilian Green Dwarf Coconut Palms Seedlings With Reduced Chemical Fertilization

**DOI:** 10.3389/fpls.2021.649487

**Published:** 2021-10-15

**Authors:** Aline Figueiredo Cardoso, Ediane Conceição Alves, Sidney D. Araújo da Costa, Alessandra Jackeline Guedes de Moraes, Dalton Dias da Silva Júnior, Paulo Manoel Pontes Lins, Gisele Barata da Silva

**Affiliations:** ^1^Plant Protection Laboratory, Institute of Agrarian Sciences, Federal Rural University of Amazon (UFRA), Belém, Brazil; ^2^Federal University of Amazonas (UFAM), Education, Agriculture and Environment Institute, Humaitá, Brazil; ^3^Sococo S.A Agroindústrias da Amazônia, Belém, Brazil

**Keywords:** *Bacillus cereus*, coconut palm, sustainable agriculture, growth promotion, PGPR

## Abstract

Coconut production in the Amazon requires the knowledge and development of sustainable technologies to alleviate the detrimental effects of inorganic chemical fertilizers and intensive farming practices. In this study, we investigated the effects of plant growth-promoting rhizobacteria (PGPR) isolated from coconut seedlings on nutrient use efficiency (NUE) and physiological mechanisms related to biomass accumulation of seedlings grown with reduced inorganic fertilizer levels. Of the 96 PGPR isolates tested on rice plants, the isolate *Bacillus cereus* (UFRABC40) was selected, as it resulted in the most significant gain in growth variables. In a commercial coconut tree nursery, we subjected seedlings to two treatments, both with seven replications: control 100% NPK chemical fertilizer (CF) and *B. cereus* + 50% NPK CF. The results indicated that the inoculation increased phytohormone levels [190% indole acetic acid (IAA), 31% gibberellic acid GA_3_, and 17% gibberellic acid GA_4_] and leaf gas exchange [48% by assimilation of CO_2_ (*A*), 35% stomatal conductance to water vapor (*gs*), 33% transpiration, and 57% instantaneous carboxylation efficiency] in leaves. Furthermore, growth parameters (shoot, root, and total dry weight, height, and diameter) and macro- and micronutrient levels (95% N, 44% P, 92% K, 103 Ca, 46% Fe, 84% B) were improved. Our results show the potential ability of strain *Bacillus cereus* UFRABC40 to promote the growth performance of coconut seedlings under decreased application of inorganic fertilizers. The application of microbial-based products in coconut seedling production systems improves plants’ physiological performance and the efficiency of nutrient use.

## Introduction

The cultivation of coconut trees is of great economic and social importance due to the value generated by coconut production. According to FAO (2018), Indonesia is the world’s largest coconut producer, followed by the Philippines, India, Sri Lanka, and Brazil. Production in Brazil occupies an area of 216 hectares, yielding approximately 2 million tons coconuts (IBGE, 2019), 1.5 million of which are obtained from green dwarf and hybrid plants (Sindcoco, 2017). The Amazon region produces 11% of the country’s coconut yield; of this, 10% (200,000 tons) comes from the state of Pará. The coconut seedlings are the first stage affecting the productivity of the perennial plant, which has a mean production-life of 40 years. Green dwarf coconut seeds have a low germination rate, and their seedlings have low vigor and quality primarily due to the incidence of leaf spots (Rabelo et al., 2006; Vinodhini and Deshmukh, 2017).

The global demand for food has resulted in large use of CFs to attain maximum agricultural efficiency. According to Wang and Li (2019), only 50% of N from fertilizer is absorbed by crops. Moreover, it has been estimated that up to 7 million tons of P per year will be used in phosphate fertilizers by 2050 (Mogollon et al., 2018). The excessive and incorrect use of fertilizers damages the environment via leaching, runoff, and erosion (Good and Beatty, 2011; Savci, 2012; Conijn et al., 2018). It also leads to changes in the soil’s physical, chemical, and microbiological characteristics (Blanco- Blanco-Canqui and Schlegel, 2013). The low efficiency of synthetic fertilizers is related to nutrient loss via leaching and evaporation to the atmosphere (Tilman, 1998; Gyaneshwa et al., 2002). Thus, the efficient use of synthetic fertilizers is important for both productivity and environmental protection (Paungfoo-Lonhienne et al., 2019). Furthermore, technologies that decrease the adverse effects of CFs on soil microbiota while promoting crop growth and productivity should be investigated.

The use of rhizobacteria in plant production can promote growth (Gange and Gadhave, 2018), and some genera such as *Pseudomonas* sp. and *Bacillus* sp. have been shown to promote the growth of coconut seedlings (George et al., 2018). Rhizobacteria can alter anatomical characteristics and improve photosynthetic, hormonal, and nutritional performance (Glick et al., 1999; Lucy et al., 2004; Lwin et al., 2012; Samaniego-Gámez et al., 2016). They also stimulate the synthesis of phytohormones such as indole acetic acid (IAA) and gibberellins that promote root and shoot growth (Pahari and Mishra, 2017). Rhizobacteria also optimize the use of CFs and are considered a sustainable technology (Angulo et al., 2020). They include N_2_ fixers, phosphorus, and potassium solubilizers (Bhardwaj et al., 2014). In rice, a 50% reduction in N and P fertilization resulted in better nutrient absorption and chlorophyll content (Naher et al., 2018). Additionally, the use of *Bacillus amyloliquefaciens* combined with 50% CF changed the hormonal behavior of oil palm seedlings in Amazonian climatic conditions, increasing IAA levels by 66%, shoot dry matter by 110%, and root dry matter by 123% and improving macro- and micronutrient uptake (Lima et al., 2020).

Coconut production in the Amazon requires knowledge and sustainable technology development to counteract the negative impacts of CF dependency. Access to the diverse range of microorganisms associated with plants and soil in the Amazon biome may improve bioinoculant production. Bioinoculant production is a process sensitive to both biotic and abiotic factors.

Therefore, this study aimed to evaluate the effects of plant growth-promoting rhizobacteria (PGPR) inoculation on coconut seedlings growth by investigating the physiological and nutritional mechanisms in seedlings grown under low CF conditions.

## Materials and Methods

The experiment was conducted in a coconut tree seedling nursery, Santa Isabel do Pará-Brazil (1°13′26″ S, 48°02′29″ W).

### Isolation of Bacterial Strains

Six soil samples containing roots (100 g) were collected from an 8-year-old dwarf coconut plantation in Brazil. Each sample was divided into portions of 10 g to obtain the isolates. Each soil sample (10 g) was diluted in 50 mL of sterile distilled water and agitated for 30 min. An aliquot of 20 μL was then separated from the original suspension and diluted in 80 μL (10^–3^). Next, an aliquot of 50 μL was separated from the concentrated suspension and seeded into three 9-mm Petri plates containing 10 mL of culture medium (per liter: g of sucrose, 8 g of hydrolyzed acid casein, 4 g of yeast extract, 2 g of K_2_HPO_4_, 0.3 g of MgSO_4_, and 15 g of agar) (Kado and Heskett, 1970). Plates were subsequently incubated at 27°C ± for 12 h. After incubation, colonies with different colors, borders, and morphology in the same plate were isolated and streaked into a new plate containing culture medium (Kado and Heskett, 1970). These plates were incubated for the same time and temperature as described above, followed by bacterial isolate purification. The bacteria were collected in microtubes containing distilled and sterile water and kept at 5°C in a refrigerator.

### Selection of Isolates

The selection of growth-promoting isolates was carried out on rice plants, and subsequently, their interaction with coconut seedlings was tested according to the method described by de Castro et al. (2020).

Briefly, rice seeds (10 g) were inoculated with 20 mL of bacterial suspension obtained from the culture growth in liquid medium to 10^8^ CFU⋅mL^–1^ (Kado and Heskett, 1970) and kept under agitation at 114 rpm at 27°C for 24 h (Filippi et al., 2011). The experimental design consisted of 97 treatments (96 rhizobacteria isolates and a control) with three replicates each in a greenhouse. Twenty-one days after germination, plants were evaluated for root and shoot length (LR and LS, respectively) and total biomass (TDM). Analysis of variance was performed for all variables, followed by a comparison of means using the Scott–Knott test (*p* < 0.05). The R40 isolate (Supplementary Material) resulted in superior growth parameters compared with all other treatments; therefore, it was subjected to *in vitro* biochemical tests and selected for subsequent testing with green dwarf coconut seedlings from Brazil.

#### Identification of Bacterial Isolate

The R40 isolate was cultured in culture medium 523 (Kado and Heskett, 1970) for 24 h at 28°C. Two inoculation loops were added to a microtube containing 1 mL of extraction buffer (Tris-HCl 1x). Then, DNA extraction was performed according to the method described by Mariano and Silveira (2005). The R40 isolate was identified using the 16S rDNA region gene and 27F (5′-AGAGTTTGATCMTGGCTCAG-3′) and 1492R (5′ACCTTGTTACGACTT-3′) primers (Lane et al., 1985). The PCR amplification reaction was composed of 1x Master Mix 2x (Promega) (0.05 U μL^–1^ Taq DNA polymerase, 4 mM MgCl_2_ reaction buffer, 0.4 mM of each DNTP; Promega Corporation, Madison, WI, United States), 10 μM of each primer, and 50 ng DNA. Amplification of the 16S rDNA region was performed in a thermal cycler (MasterCycler Nexus, Eppendorf, Hamburg, Germany) with the following steps: initial denaturation at 94°C for 4 min; 25 cycles of 94°C for 1 min, 55°C for 1 min, and 72°C for 1 min; and a final extension at 72°C for 7 min. Reactions were purified using 5 μL of PCR product plus 2 μL Exo-SAP enzyme (Exonuclease). Samples were purified via a thermal cycler, performed at 37°C for 4 min, followed by an incubation period at 80°C for 1 min to inactivate both enzymes irreversibly. After the purification reaction, sequencing was carried out in an automated sequencer (ABI3730) at the Laboratory of Bioinformatics and Evolutionary Biology, Federal University of Pernambuco (LABBE-UFPE).

DNA sequence analysis and assembly of the R40 isolate contigs were performed using the Staden Package (Staden et al., 1998). The nucleotide sequence of the UFRABC40 bacteria was compared with the isolate sequences available in the National Center for Biotechnology Information (NCBI) database using the BLASTn software^1^. Afterward, all sequences were aligned (MEGA). Bayesian inference (IB) analysis was performed by means of Mr. Bayes v. 3.2.6 (Ronquist et al., 2012) implemented in CIPRES^2^ using the best nucleotide replacement model. This was selected according to Aikake’s Information Criterion (AIC) through Mr. Modeltest 2.3 (Nylander, 2004) using 1,000,000,000 generations of Markov Chain Monte Carlo (MCMC) with sampling every 1,000 and 10,000 generations. Identification of access and phylogenetic trees was obtained by comparing the selected strain with the reference strains using 29 reference accessions to identify the selected strain (R40). Identifying a bacterial isolate using 16S rRNA was used to identify the strain selected with the strain of greatest homology. Subsequent probabilities were calculated after discarding the first 25% of the generations. All trees obtained from individual genes and concatenated through the IB method were visualized through the Fig Tree 1.4.1 software^3^.

#### Biochemical Tests

##### Indole Acetic Acid Production

The R40 isolate was grown in a Luria Bertani (LB) medium under 100 rpm agitation and incubated at 28°C for 78 h. Subsequently, 3 mL of the suspension was centrifuged at 4°C for 10 min at 4,000 rpm (Moustaine et al., 2017). Then, 90 μL of the supernatant and 60 μL of the Salkowski reagent were placed in a microtube and incubated in the dark for 30 min to determine if a change in mean media color occurred (Gordon and Weber, 1951).

##### Production of Siderophores

The R40 isolate was inoculated into test tubes containing a 10 mL Tryptic Soybean Broth (TSB) (1:10 diluted) medium (3 g in 1,000 mL distilled water) and incubated at 28°C under agitation at 114 rpm for 24 h. Subsequently, tubes containing the bacterial suspension were centrifuged for 10 min at 12,000 rpm. Then, 1 mL of the supernatant was transferred into another tube containing 1 mL of the blue chrome S (BCS) solution. Fifteen minutes after mixing, if siderophores were produced, the dark blue mixture turned yellow (Schwyn and Neilands, 1987).

##### Phosphate Solubilization

The R40 isolate was grown in an NBRIP growth medium containing 10 g glucose, 2.5 g Ca_3_(PO_4_), 25 g MgCl_2_W_6_H_2_O, 0.25 g MgSO_4_W_7_H_2_O, 0.2 g KCl, and 0.1 g (NH_4_)_2_SO_4_ (Nautiyal, 1999), at a pH of 7.0, and with the addition of 1.5% agar in triplicate. The plates were incubated for 14 days at 28°C; the presence of a halo was indicative of phosphate solubilization.

### Evaluating R40 Isolate Ability to Promote Growth Coconut Seedlings

#### Coconut Seeds Preparation

In a coconut tree nursery, the coconut seeds were sown in wooden boxes (30 cm high, 2 m wide, and 10 m long) containing coconut fiber and moistened daily for 90 days. The chemical characterization of the coconut fiber substrate (Golden Mix type 4 – AMAFIBRA^®^) was as follows: 0.086 g kg^–1^ N, 0.264 g kg^–1^ P, 0.580 g kg^–1^ K, 0.128 mg kg^–1^ Ca, 0.447 mg kg^–1^ MgO, 272.86 mg kg^–1^ S, 42.25 mg Na, 0.703 mg L^–1^ B, 0.12 g kg^–1^, copper Cu, 0.5 mg kg^–1^ Fe, 0.6 mg kg^–1^ Mn, 0.78 mg kg^–1^ Zn, and 92.43% organic matter (OM).

#### Coconut Seedling Preparation

Seedlings with 15 cm tall and two leaves were transplanted to polyethylene bags (40 × 40 × 40 cm) containing 7.5 kg/bag of coconut fiber (50% moist). Chemical fertilization was performed 30 days after transplanting (DAT) with 3 g urea, 40 g simple superphosphate (18% P_2_O_5_), 10 g potassium chloride (60% K_2_O), and 5 g magnesium oxide (30% Mg) (Lins and Viégas, 2008).

#### Evaluation of Coconut Seedling Growth

For the establishment of treatments, the recommendation of commercial fertilization was followed. Thus, the control treatment is characterized as 100% chemical fertilization (CF) and without the use of bioinoculant. The control treatment used standard CF (as described in the previous section) applied at 90 and 150 DAT. The treatment with rhizobacteria comprised inoculation with a suspension of R40 isolate (10^8^ CFU) + 50% standard CF at 90 DAT. The bacterial strain was inoculated by applying 300 mL plant^–1^ of a suspension at 10^8^CFU⋅mL^–1^ through watering at 40 and 70 DAT. Biometrics, gas exchange, hormone levels, and nutrient levels were evaluated at 160 DAT. The experimental design was completely randomized, with 10 replications and 2 treatments.

##### Biometrics

The following biometric variables were evaluated: shoot, root, and total dry weight, height, and diameter. Additionally, the leaf area was determined from photographs using the APS Assess software version 2.0 (Lamari, 2002).

##### Leaf Gas Exchange

Gas exchange parameters were estimated using the first physiologically mature, fully expanded leaf, from apex to base, at 3 months of age. The net assimilation of CO_2_ (*A*), stomatal conductance to water vapor (*gs*), transpiration rate (*E*), and instantaneous carboxylation efficiency (*A/Ci)* were estimated between 08:00 and 11:00 am using a portable open-flow gas-exchange system (LI6400XT, LI-COR, Lincoln, NE, United States) under an external CO_2_ concentration of 400 μmol mol^–1^ of air and artificial photosynthetically active radiation (PAR) of 900 μmol of photons m^–2^ s^–1^.

##### Hormone Profile

Indole acetic acid (IAA) and gibberellic acid (GA_3_ and GA_4_) hormone levels were determined according to Munné-Bosch et al. (2011). For this, 300 mg of fresh tissue from the second leaf of each plant was stored in liquid N. The tissue material was then lyophilized and macerated in liquid N. Then, 40 mg dry mass was weighed, and 400 μL of extraction solvents (methanol:isopropyl alcohol:acetic acid; 20:79:1) was added. Samples were vortexed four times for 20 s (on ice), sonicated for 5 min, placed on ice for 30 min, and then centrifuged at 13,000 rpm for 10 min at 4°C. After centrifugation, 350 μL of supernatant was removed and transferred to another microtube. Approximately 300 μL of the extract obtained in flasks was added, and 5 μL of the mixture was injected into the NuBioMol LC/MS system (Biomolecule Analysis Center, UFV, Brazil). A chromatography column (Agilent Eclipse; Agilent Technologies, Santa Clara, CA, United States) was used (RRHD, C18 column, 50 mm × 2.1 mm, 1.8 μm) with a flow rate of 0.3 mL min^–1^ coupled to a triple quadrupole QQQ mass spectrometer (Agilent Technologies). Mass spectra were alternately negative/positive operated according to the retention time for each hormone. The generated mass spectra were processed using the MassHunter software to obtain the extracted ion chromatograms (XIC) for each transition and area values, indicating the abundance of each hormone. A curve pattern for each hormone over a concentration range from 0.1 to 300 ng mL^–1^ was used to convert the XIC area values into ng g^–1^ of plant tissue. Molecular mass spectra analysis was conducted using the Skyline software.

##### Nutritional Content

Leaf samples dried in an oven with forced air circulation at 60°C were ground. The samples were submitted to sulfuric and nitroperchloric digestion. The determination of nitrogen (N) was by distillation in Microdistillator Kjeldhal, phosphorus (P) by visible ultraviolet spectrophotometry (UV-VIS), and potassium (K), calcium (Ca), and iron (Fe) by absorption spectrometry atomic, flame modality (EAA/cham). Analysis of boron (B) was undertaken after dry digestion of the samples using the method described by Azometrinah (Malavolta et al., 1997; Carmo et al., 2000). The nutrient use efficiency (NUE) was estimated from agronomic efficiency, NUE (g DW g ^–1^) = aerial dry weight (g)/plant applied nutrient (g) (Fageria et al., 2008).

### Statistical Analysis

Differences among means for treatments were evaluated using the *t*-test (*p* < 0.05). All data were analyzed using the R software (R Core Team, 2017).

## Results

### Isolate Selection in the Plant Model

**FIGURE 1:**
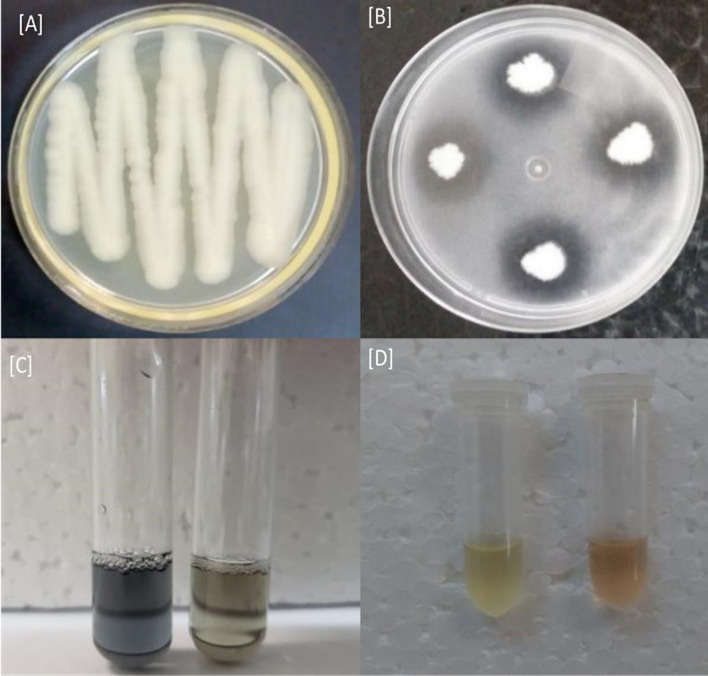
R40 isolate *in vitro* biochemical tests. **(A)** R40 isolate colony in Petri plate, **(B)** phosphate solubilization detection, **(C)** siderophore production, and **(D)** indole acetic acid (IAA) production.

The rice plants used as a model for selecting rhizobacteria showed that the R40 isolate was better for root and shoot length variables and total biomass compared with the other treatments (Table 1). The isolate R40 increased by 101% shoot length, 60% root length and 280% total biomass in comparation that is seedlings non-bioinoculation. *In vitro* biochemical tests were performed, showing that the R40 isolate was able to solubilize phosphate as proven by the halo formation around the bacterial colonies. There were also reactions indicating siderophore and IAA production when the R40 isolate was exposed to CAS solution and Salkowski’s test, respectively (Figure 1). Thus, the R40 isolate was selected to evaluate growth promotion in green dwarf coconut seedlings in Brazil under nursery conditions.

**TABLE 1:** Selection of growth-promoting rhizobacteria in rice, isolated from the rhizosphere of commercially grown green dwarf coconut trees from Santa Isabel, PA, Brazil.

Isolate	LA	LR	TDM	Isolate	CL	RL	TDM
					
	(cm)		(g)		(cm)		(g)
1	35.53b	16.16d	0.10b	51	35.26b	15.33d	0.07c
2	34.16b	20.00b	0.08c	52	36.28b	15.75d	0.08c
3	34.95b	27.75b	0.09c	53	32.73b	22.56b	0.10b
4	46.03a	13.66e	0.09c	54	31.50b	20.30b	0.10b
5	32.33c	25.91b	0.11b	55	36.35b	20.06b	0.05c
6	37.45b	21.10b	0.16ab	56	31.20b	23.63b	0.13b
7	32.16b	19.33c	0.10b	57	27.90c	20.80b	0.06c
8	33.00b	14.16e	0.09c	58	32.16b	17.93d	0.08c
9	34.6b	19.5c	0.16ab	59	28.16c	18.70d	0.07c
10	32.66b	21.53b	0.07c	60	46.30a	16.60d	0.09c
11	32.06b	21.83b	0.09c	61	28.34c	11.83e	0.09c
12	36.00b	11.00e	0.09c	62	28.13c	18.23d	0.07c
13	32.86b	20.60b	0.12b	63	31.40b	27.13ab	0.09c
14	42.33a	20.00b	0.08c	64	36.20b	23.40b	0.12b
15	41.10a	15.33d	0.12b	65	27.90c	21.13b	0.13b
16	43.00a	25.00b	0.12b	66	35.06b	24.23b	0.11b
17	37.93b	18.90d	0.10b	67	25.10c	14.50e	0.06c
18	43.66a	14.66e	0.10b	68	30.90b	27.03ab	0.13b
19	40.50a	24.53b	0.10b	69	36b	27.8a	0.1b
20	41.50a	20.46b	0.09c	70	29.03bc	22.2b	0.06c
21	32.23b	23.86b	0.15b	71	27.9c	20.8b	0.06c
22	33.70b	20.56b	0.14b	72	29bc	22b	0.09c
23	33.56b	19.26c	0.12b	73	26.8c	17d	0.13b
24	34.23b	20.16b	0.08c	74	22.76c	13.90e	0.10b
25	34.56b	23.90b	0.12b	75	24.90c	13.66e	0.07c
26	35.53b	23.30b	0.14b	76	30.60b	18.26d	0.05c
27	42.83a	16.06d	0.09c	77	28.23c	20.90d	0.13b
28	38.46b	15.03d	0.08c	78	32.63b	18.43d	0.11b
29	32.7b	22.6b	0.01d	79	27.90c	19.50d	0.08c
30	37.5b	18.1d	0.07c	80	27.70c	19.03d	0.06c
31	34.4b	17.9d	0.07c	81	35.16b	25.46b	0.14b
32	36.3b	14.5e	0.08c	82	38.13b	27.83ab	0.17ab
33	35.35b	16.20d	0.07c	83	28.60c	21.53b	0.09c
34	40.76a	13.16e	0.06c	84	35.80b	21.86c	0.15b
35	39.30b	15.03d	0.12b	85	39.40b	24.63b	0.15b
36	36.83b	18.76d	0.13b	86	36.23b	16.26d	0.08 e
37	40.60a	17.23d	0.15b	87	34.70b	19.60d	0.17ab
38	34.20b	22.16b	0.15b	88	31.80b	20.33d	0.16ab
39	35.46b	23.80b	0.17ab	89	32.26b	24.61b	0.14b
40*	49.6a	28.5a	0.19a	90	31.10b	23.36b	0.12b
41	37.33b	21.66b	0.12b	91	27.90c	20.80b	0.07 e
42	45.80a	22.60b	0.13b	92	33.10b	24.54b	0.15b
43	46.23a	21.36 b	0.15b	93	38.13b	16.66d	0.17ab
44	36.20b	19.63b	0.16ab	94	39.56b	17.40d	0.12b
45	36.93b	23.40b	0.14b	95	28.23c	21.80c	0.13b
46	30.90b	21.30b	0.08c	96	36.33b	17.4d	0.12b
47	28.33c	14.83e	0.05c	Control	24.70c	17.86d	0.05 e
48	35.50b	20.63a	0.14b				
49	31.90b	16.53d	0.08c				
50	26.80c	17.06d	0.16ab				

Same letters indicate no significant difference (SNK test, *p*< 0.05). LR, root length; LA, shoot length (determined with the aid of a millimeter rule); TDM, total biomass (g/weight of dryplant). *Isolate selected as promising for promoting growth in model plants.

### Growth-Promotion Coconut Seedlings

The R40 isolate sequence was compared in GenBank using the BLASTn tool. The isolate showed 100% identity with the genus *Bacillus* (ATCC14579T). Based on the construction of the phylogenetic tree from 29 accesses, it was possible to identify the isolate as *B. cereus*. The sequence was deposited in GenBank as *B. cereus* (UFRABC40) with accession number MN393059 (Supplementary Table 1 and Supplementary Figure 1).

**FIGURE 2:**
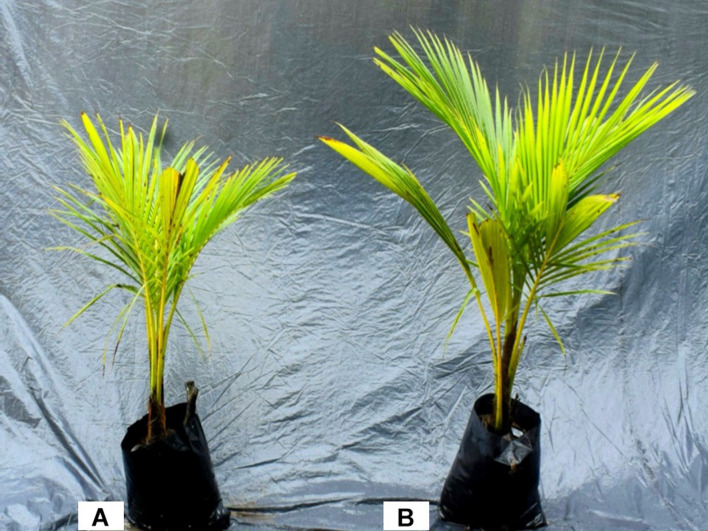
Green dwarf coconut seedlings from Brazil. **(A)** Control coconut seedlings with 100% chemical fertilization and **(B)** coconut seedlings inoculated with *Bacillus cereus* and 50% chemical fertilization.

**FIGURE 3:**
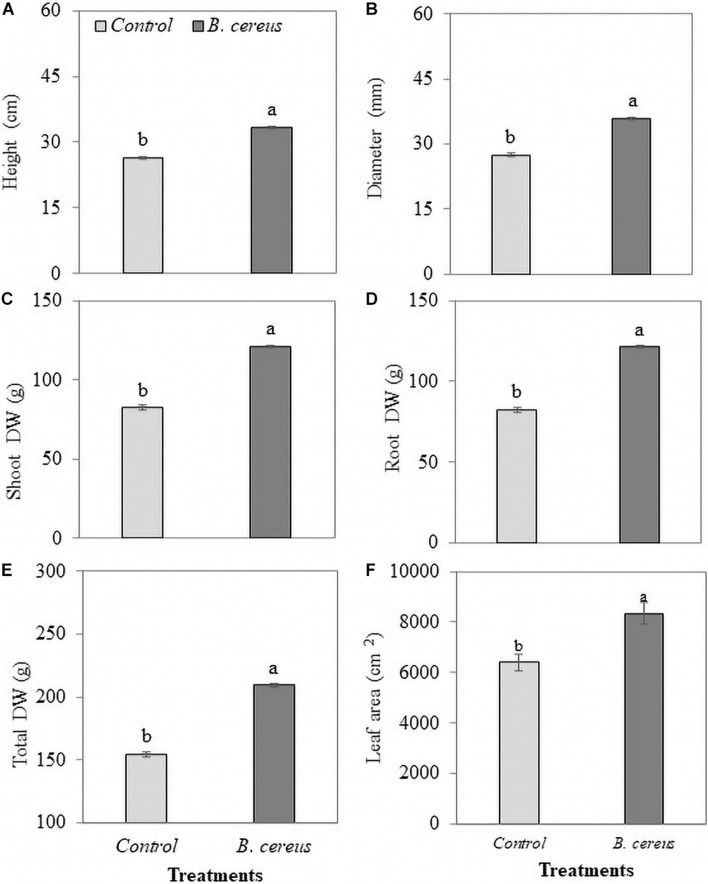
Biometrics of green dwarf coconut seedlings from Brazil (uninoculated and inoculated with *B. cereus*). **(A)** Height, **(B)** stem diameter, **(C)** shoot dry matter, **(D)** root dry matter, **(E)** total dry matter **(E)**, and **(F)** leaf area in plants with 100% chemical fertilization (control) and plants inoculated with *Bacillus cereus* with 50% chemical fertilization. The same letters indicate no significant difference (*t*-test, *p* < 0.05).

The inoculation of strain *B. cereus* promoted the growth of coconut seedlings even in the presence of lower levels of chemical fertilizers (Figure 2). The application of *B. cereus* significantly increased shoot dry weight (47%), root dry weight (122%), total dry weight (35%), height (26%), and diameter (30%) compared with the control treatment (Figure 3).

**FIGURE 4:**
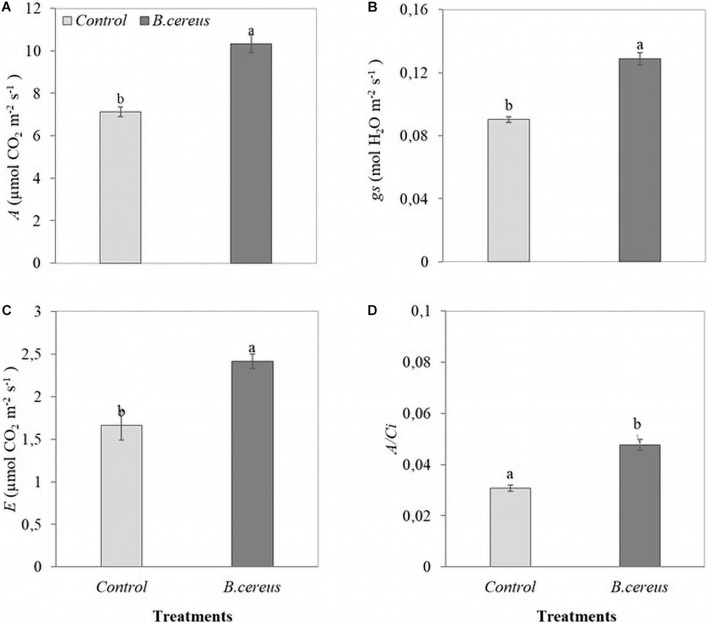
Leaf gas exchange in green dwarf coconut seedlings. **(A)** The net assimilation of CO_2_ (*A*), **(B)** stomatal conductance to water vapor (*gs*), **(C)** transpiration (*E*), and instantaneous carboxylation efficiency (*A/Ci*) in plants with 100% chemical fertilization (control) and plants inoculated with *Bacillus cereus* with 50% chemical fertilization. The same letters indicate no significant difference (*t*-test, *p* < 0.05).

**FIGURE 5:**
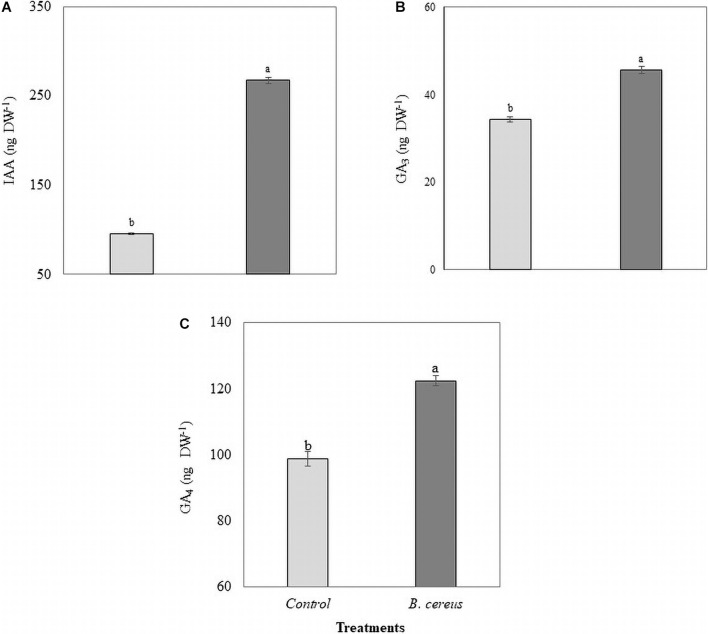
Phytohormone quantification in coconut seedlings. **(A)** Indoleacetic acid (IAA), **(B)** gibberellic acid GA_3_, and **(C)** gibberellic acid GA_4_ in plants with 100% chemical fertilization (control, T1) and plants inoculated with *Bacillus cereus* with 50% chemical fertilization (T2). The same letters indicate no significant difference (*t*-test, *p* < 0.05).

Gas exchange was also influenced by inoculation with *B. cereus* from the third month of age in Brazilian green dwarf coconut tree seedlings. The nursery trial results indicated a maximum increase of 48% in *A*, 35% in *gs*, 33% in *E*, and 57% in *A/Ci* in plants inoculated with *B. cereus* compared with the uninoculated control (Figure 4). *B. cereus* inoculation led to an increase of 190% in IAA, 31% in GA_3_, and 17% in GA_4_ in coconut seedlings compared with the uninoculated control (Figure 5).

The *B. cereus* application significantly increased macronutrients and micronutrients in coconut plants by 95%, 44%, 82%, 103%, 46%, and 84% for N, P, K, Ca, Fe, and B, respectively (Table 2), compared with the control. Treatment with *B. cereus* shows greater efficiency in the use of nutrients (Table 3).

**TABLE 2:** Nutritional content in the shoots of green dwarf coconut seedlings from Brazil.

Treatments	N	P	K	Ca	Fe	B
		
	(g/Dry leaf weight)			(mg/Dry leaf weight)		
Control	0.71^b^	0.09^b^	1.28^b^	0.33^b^	10.29^b^	1.21^b^
*B. cereus*	1.39^a^	0.13^a^	2.33^a^	0.67^a^	15.02^a^	2.23^a^

*Control treatment (100% chemical fertilization) and inoculated with *Bacillus cereus* combined with 50% chemical fertilization. The same letters indicate no significant difference (*t*-test, *p* < 0.05).*

**TABLE 3:** Nutrient use efficiency (NUE) of green dwarf coconut seedlings from Brazil.

Treatments	N	P	K	Ca	Fe	B
	
	NUE (g DW g ^–1^)					
Control	8.7^b^	0.7^b^	2.5^b^	107.2^b^	27.5^b^	19.6^b^
*B. cereus*	25.9^a^	2.0^a^	7.3^a^	320.6^a^	82.1^a^	56.6^a^

*Dry weight (DW). Control treatment (100% chemical fertilization) and inoculated with *Bacillus cereus* combined with 50% chemical fertilization. The same letters indicate no significant difference (*t*-test, *p* < 0.05).*

## Discussion

The rhizobacteria *B. cereus* promoted the growth of green dwarf coconut seedlings. Bacterial inoculation induced changes in the metabolism of coconut tree seedlings, by stimulating hormonal modulation, and photosynthetic performance and efficient use of nutrients, which ultimately resulted in a greater growth of coconut tree seedlings. These results may be due to the increase in IAA concentrations derived from the enhanced production by *B. cereus* strain (as shown in Figure 1) or due to the regulation of its biosynthesis in the plants (Figure 5). Indole acetic acid is responsible for modulating the differentiation and elongation of lateral roots, as well as increasing the number of root hairs, therefore promoting greater nutrient absorption (Costa et al., 2015; Cassán et al., 2020). Combined with the positive effects of IAA, it is possible that bioinoculation has reduced ethylene levels in the roots by the activity of the enzyme 1-aminocyclopropane-1-carboxylic acid (ACC) deaminase. This enzyme regulates ethylene synthesis by cleaving its acid precursor, ACC (Belimov et al., 2004; Siddikee et al., 2011), thus decreasing the negative effects of ethylene on growth and allowing the development of a better root system in inoculated plants (Glick, 2012). *Bacillus cereus* inoculation resulted in the more efficient use of macro- and micronutrients. A significant positive effect on the levels of the seven nutrients evaluated was observed (Tables 2, 3). Recommendations for the use of CFs are based on soil analysis and the mechanisms of macro- and micronutrient loss, such as volatilization, leaching, and adsorption, resulting in low absorption by the plant (Biswas et al., 2000; Ahmad et al., 2019). These root regions have a high influx of available ions, resulting in greater water and nutrient absorption. Root system changes induced by microorganisms improve NUE. This phenomenon has been reported in the interaction between banana and the bacterial strains *Pseudomonas fluorescens* Ps006 and *Bacillus amyloliquefaciens* Bs006 (Gamez et al., 2018) resulting in greater plant biomass. Another mechanism involved in the NUE of biostimulated coconut plants is the mineralization rate of the coconut fiber substrate by bioinoculant enzymatic activity, resulting in increased nutrient availability.

For N, *B. cereus* may make N available from the organic N contained in the coconut fiber via ammonium and nitrite oxidation. This was described by Di Benedetto et al. (2016), who found that *Pseudomonas* and *Bacillus* strains were able to oxidize ammonia to NO_2_^–^ ions (nitrosification) and then to NO_3_^–^ ions (nitrification). In a study with *Triticum aestivum*, the inoculation of *Bacillus megaterium* SNji (BmeSNji) and *Azospirillum brasilense* 65B (Abr65B) provided greater availability of nutrients to plants, such as N, from decomposition of organic matter resulting in greater accumulation of biomass in plants (Nguyen et al., 2019).

Coconut plants inoculated with *B. cereus* also had enhanced K uptake. This might be explained by its ability to produce organic acids that act in the mineralization of K present in the coconut fiber substrate, making K^+^ ions available for plant absorption. According to Sheng and He (2006), the *Bacillus edaphicus* NBT strain and its mutants can chelate metals and mobilize K from K-containing minerals using organic acids such as citric, oxalic, tartaric, and succinic.

Solubilization of P by rhizobacteria requires the production of phosphatases and phytases that mineralize the organic material by esters and H_3_PO_4_ anhydride hydrolysis (Tabatabai, 1994; Nannipieri et al., 2011). In maize, *Pseudomonas plecoglossicida* (PSB5) inoculation increased P production by 18% and its total absorption by 46%. This was due to the increased activity of enzymes such as dehydrogenases and phytases (Kaur and Reddy, 2013). In this study, *B. cereus* was able to solubilize P *in vitro*, and plants inoculated with this strain showed higher levels of P than control ones. As observed *in vitro*, *B. cereus* can produce siderophores, which are low molecular weight iron-chelating compounds with a great affinity and selectivity for binding and forming a Fe complex (III), reducing Fe^3+^ to Fe^2+^ (Hider and Kong, 2010; Fukushima et al., 2013). In mustard, the capacity of *Bacillus* sp. PZ-1 to produce siderophores resulted in higher Fe levels available for the plant (Yu et al., 2017).

Boron is a micronutrient essential for plant growth and development, and coconut plants have high B requirements (Moura et al., 2013). Brazilian Amazonian soils are generally deficient in B, and appropriate CF use is critical to avoid B deficiency. However, the application of excess exogenous B can easily be lethal to plants. Boron is not described as an essential nutrient for PGPR growth; however, in *Arthrobacter nicotinovorans* strain C, phenylboronic acid (PBA) catabolism was demonstrated, releasing B as orthoboric acid [B(OH)_3_] (Negrete-Raymond et al., 2003). Rhizobacterium-mediated B availability to plants occurs through the production of organic acids in the rhizosphere region, resulting in medium acidification and pH decrease, the latter being the main limiting factor in B availability (Deubel et al., 2000; Turan et al., 2006). Concomitant B uptake by the plant occurs through the mass flow from transpiration (Alpaslan and Gunes, 2001). Therefore, the increased transpiration rate provided by PGPR inoculation can influence B absorption (Mayak et al., 2004; Dodd and Pérez-Alfocea, 2012). In the present study, plants inoculated with *B. cereus* had almost twice the accumulated B in the shoots and twice the transpiration rate compared with the control. According to our analysis, this may be due to the enzymatic activity of *B. cereus* on the coconut fiber substrate that contained 70.35 ppm of B. The positive relationship between P and B absorption found in plants inoculated with *Bacillus* sp. has been recorded in canola; *Bacillus* improved B and P availability by 37% and 30%, respectively, in native soil (Samreen et al., 2019). These results are like ours, obtained with *B. cereus* coconut seedlings (Table 2). The increasing use of CFs especially with NPK and B is rapidly making them polluting agents. When not absorbed by the plants, they are leached and deposited in watercourses, or immobilized and accumulated in the soil. Implementing microbial technology in coconut seedlings tree production systems improves the physiological performance and NUE of plants, reducing the need for CFs.

In this study, the increases in the root system induced by *B. cereus* provided coconut seedlings with a greater possibility of absorption and translocation of nutrients, contributing to a greater growth in the aerial part (Amir et al., 2005). The increase in the aerial part growth and leaf expansion promoted by *B. cereus* can be attributed to the greater synthesis of gibberellins, according to the results obtained in this study (Figure 3). Increased active gibberellin concentration in the leaf tissue was stimulated by the activity of *Azospirillum* spp. because this microorganism promoted a significant synthesis and consequent increase in the concentration of this phytohormone (Lucangeli and Bottini, 1997; Piccoli et al., 1997; Cassán et al., 2001). Similar results were observed in alder (*Alnus glutinosa*) plants, where the use of *Bacillus* sp. enhanced the production of several isomers of gibberellins (GA_1_, GA_3_, GA_4_, and GA_20_) that were responsible for leaf area expansion and increased leaf emission rate (Gutiérrez-Mañero et al., 2001; Chauhan et al., 2015). Larger leaf areas resulted in a higher biomass accumulation in *B. cereus* inoculated plants.

This leaf modulation promoted by the microorganisms led to increased light capture and, consequently, increased CO_2_ assimilation. The increased CO_2_ input is due to a larger stomatal opening, verified in the present study, allowing greater CO_2_ diffusion, and reducing stomatal resistance (Flexas et al., 2012; Zhang et al., 2017). *B. cereus* regulates most of the Rubisco carboxylation, the electron transport rate, and increases the supply of ATP and NADPH molecules for photosynthesis (Shi et al., 2010), increasing carbon fixation and, consequently, increasing carbohydrate production as observed in the improved growth parameters of *B. cereus* inoculated plants.

## Data Availability Statement

The datasets presented in this study can be found in online repositories. The names of the repository/repositories and accession number(s) can be found below: https://www.ncbi.nlm.nih.gov/genbank/, MN393059.

## Author Contributions

AC was present in all study assays and was responsible for fieldwork, statistical analysis of data, interpretation of results, and writing the manuscript. SC was present in all study assays and was responsible for fieldwork. DS, AM, EA, and PL were responsible for statistical analysis, analysis of economic indicators, and interpretation of results and assisted in writing and revising the manuscript. GS was responsible for project management, guiding the students through all stages of the process and also responsible for the verification and monitoring of field data and writing the manuscript. All authors contributed to the article and approved the submitted version.

## Conflict of Interest

The authors declare that the research was conducted in the absence of any commercial or financial relationships that could be construed as a potential conflict of interest.

## Publisher’s Note

All claims expressed in this article are solely those of the authors and do not necessarily represent those of their affiliated organizations, or those of the publisher, the editors and the reviewers. Any product that may be evaluated in this article, or claim that may be made by its manufacturer, is not guaranteed or endorsed by the publisher.

## Footnotes

1
https://www.ncbi.nlm.nih.gov

2
https://www.phylo.org/portal2/home.action

3
http://tree.bio.ed.ac.uk/software/figtre

## Supplementary Material

Supplementary Figure 1 Phylogenetic trees analysis of R40 (UFRABC40). Major Bacillus cereus clades and groups are indicated comparing the selected strain with the reference strains.

Supplementary Table 1 Identification of access and phylogenetic trees obtained by comparing the selected strain (R40) with the reference strains.
