# Supplementary figures and images for: Bacillus cereus Improves Performance of Brazilian Green Dwarf Coconut Palms Seedlings With Reduced Chemical Fertilization
Source: Front Plant Sci. 2021 Oct 15;12:649487. doi: 10.3389/fpls.2021.649487 (PMC8553962; doi:10.3389/fpls.2021.649487)

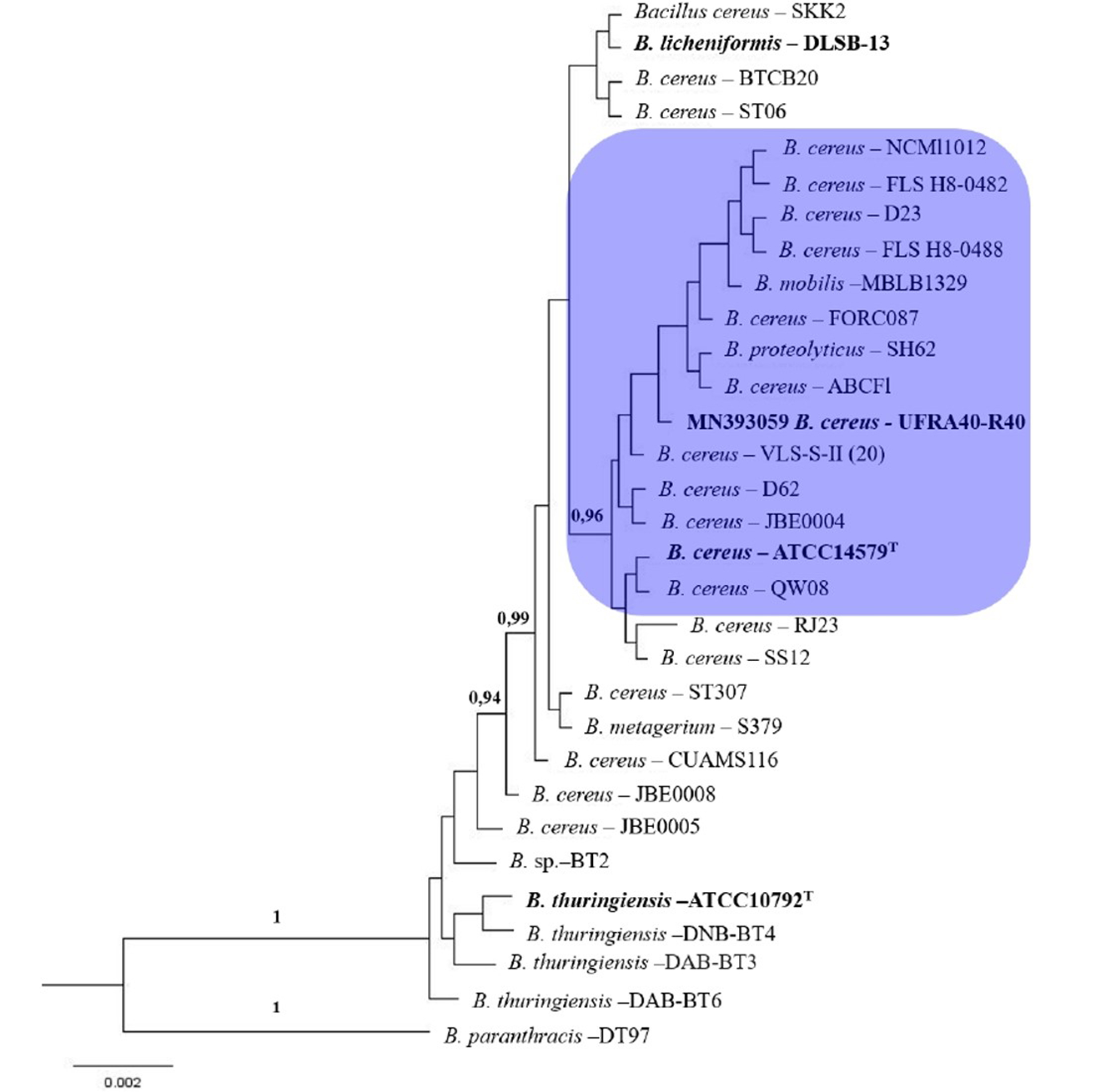

Supplement: Supplementary Figure 1 — Phylogenetic trees analysis of R40 (UFRABC40). Major Bacillus cereus clades and groups are indicated comparing the selected strain with the reference strains. [file Image_1.jpeg]
