# Supplementary material for: Bacillus cereus Improves Performance of Brazilian Green Dwarf Coconut Palms Seedlings With Reduced Chemical Fertilization
Source: Front Plant Sci. 2021 Oct 15;12:649487. doi: 10.3389/fpls.2021.649487 (PMC8553962; doi:10.3389/fpls.2021.649487)
Supplement: Supplementary Table 1 — Identification of access and phylogenetic trees obtained by comparing the selected strain (R40) with the reference strains. [file Table_1.doc]

**Supplementary Table 1**: **Identification of access and phylogenetic trees obtained by comparing the selected strain (R40) with the reference strains.**

| **Identification** | **Name** | **Strain** | **Accession** | **Similarity** |
| --- | --- | --- | --- | --- |
| **1** | ***Bacillus cereus*** | **UFRA40 - R40** | **MN393059.1** | - |
| **2** | ***Bacillus cereus*** | **ATCC14579^T^** | **AF290547.1** | **100%** |
| 3 | *Bacillus cereus* | BTCB20 | MK118714.1 | 99,57% |
| 4 | *Bacillus cereus* | ST06 | MF496242.1 | 99,79% |
| 5 | *Bacillus cereus* | NCIB 40112 | AJ310100.1 | 99,57% |
| 6 | *Bacillus cereus* | D23 | DQ923480.1 | 99,43% |
| 7 | *Bacillus mobilis* | MBLB1329 | MK280737.1 | 99,42% |
| 8 | *Bacillus cereus* | FORC087 | NZ_CP029454.1 | 99,45% |
| 9 | *Bacillus cereus* | SH62 | EU374156.1 | 93,74% |
| 10 | *Bacillus cereus* | VLS-S-II (20) | MH475929.1 | 99,79% |
| 11 | *Bacillus cereus* | D62 | DQ923487.1 | 99,79% |
| 12 | *Bacillus licheniformis* | DLSB-13 | MK795391.1 | 99,79% |
| 13 | *Bacillus cereus* | QW08 | MK760071.1 | 99,79% |
| 14 | *Bacillus cereus* | RJ23 | KC990812.1 | 99,35% |
| 15 | *Bacillus cereus* | SS12 | KM001604.1 | 98,68% |
| 16 | *Bacillus cereus* | ST307 | EU350369.1 | 99,79% |
| 17 | *Bacillus megaterium* | S379 | KY681799.1 | 94,51% |
| 18 | *Bacillus cereus* | CUAMS116 | MN093299.1 | 99,79% |
| 19 | *Bacillus sp.* | BT2 | LC055678.1 | 94,75% |
| 20 | *Bacillus thuringiensis* | ATCC10792^T^ | AF290545.1 | 99,79% |
| 21 | *Bacillus thuringiensis* | DNB-BT4 | AM293343.1 | 99,57% |
| 22 | *Bacillus thuringiensis* | DAB-BT3 | EU124379.1 | 99,43% |
| 23 | *B. thuringiensis* | DAB-BT6 | AM293344.1 | 99,50% |
| 24 | *Bacillus cereus* | FLS H8-0482 | KU198623.1 | 99,50% |
| 25 | *Bacillus cereus* | FSL H8-0488 | KU198624.1 | 99,57% |
| 26 | *Bacillus cereus* | JBE0008 | FJ982659.1 | 99,65% |
| 27 | *Bacillus cereus* | JBE0005 | FJ982657.1 | 99,65% |
| 28 | *Bacillus cereus* | JBE0004 | FJ982654.1 | 99,79% |
| 29 | *Bacillus cereus* | ABCFI | MN121339.1 | 99,79% |
| 30 | *Bacillus cereus* | kk2 | JX470956.1 | 99,57% |
